# Using max standardized uptake value from positron emission tomography to assess tumor responses after lung stereotactic body radiotherapy for different prescriptions

**DOI:** 10.1002/acm2.12453

**Published:** 2018-09-14

**Authors:** Meisong Ding, William Zollinger, Robert Ebeling, David Heard, Ryan Posey

**Affiliations:** ^1^ Department of Radiation Oncology Tulane University Medical Center New Orleans LA USA; ^2^ Northeast Louisiana Cancer Center Monroe LA USA

**Keywords:** computed tomography (CT), maximum standardized uptake value (SUV), positron emission tomography (PET), stereotactic body radiotherapy (SBRT), tumor responses

## Abstract

**Purpose:**

To retrospectively investigate tumor responses of lung SBRT patients for different prescriptions. To analyze the relation between optimal biologically equivalent dose (BED) and tumor responses.

**Methods and Materials:**

Tumor responses after lung SBRT were compared by examining 48 treatments used four prescriptions. This study used simplified tumor response criteria: (a) Complete Response (CR) — post max SUV (SUV_post_) after SBRT in the treated tumor region was almost the same as the SUVs in the surrounding regions; (b) Partial Response (PR) — SUV_post_ was smaller than previous max SUV (SUV_pre_), but was greater than the SUVs in the surrounding regions; (c) No Response (NR) — SUV_post_ was the same as or greater than SUV_pre_. Some SUV_post_ reported as mild or favorable responses were classified as CR/PR. BED calculated using *α*/*β* of 10 Gy were analyzed with assessments of tumor responses for SBRT prescriptions.

**Results:**

For the prescriptions (9 Gy × 5, 10 Gy × 5, 11 Gy × 5, and 12 Gy × 4) historically recommended by RTOG, we observed that higher BED_10_ and lower tumor volume would achieve a higher complete response rate. The highest complete response rate was observed for smallest tumor volume (PTV_ave_ = 6.8 cc) with higher BED_10_ (105.6) of 12 Gy × 4 prescription. For 11 Gy × 5 prescription, the BED_10_ (115.5) was the highest, but its complete response rate (58%) was lower than 79% of 12 Gy × 4 prescription. We observed the PTV_ave_ of 11 Gy × 5 prescription was more than double of the PTV_ave_ of 12 Gy × 4 prescription. For the same lung SBRT prescription (BED_10_ > 100) earlier staging tumor had more favorable local control.

**Conclusion:**

We demonstrated post max SUV read from PET/CT could efficiently and accurately assess tumor response after lung SBRT. Although SBRT with prescriptions resulting in a BED_10_ > 100 experienced favorable tumor responses for early staging cancer, escalation of BED_10_ to higher levels would be beneficial for lung cancer patients with later staging and larger volume tumors.

## 1 INTRODUCTION

Positron Emission Tomography (PET) is an imaging technique that provides unique information about the molecular and metabolic changes associated with disease. The technology has existed for more than 50 years^1^ but has only been used clinically after 18F‐fluorodeoxyglucose (FDG) and other isotopes were synthesized 30 years ago.^2^, ^3^, ^4^ In 2000 the Food and Drug Administration approved the use of 18F‐FDG to assist in evaluation of malignancy in patients with known or suspected abnormalities found by other testing methods. In 2008 a multidisciplinary expert panel of oncologists, radiologists, and nuclear physicians recommended the use of 18F‐FDG PET in oncology practice and determined the suitability of 18F‐FDG PET in managing various forms of cancer.^5^ 18F‐FDG PET has been recognized as an important tool for the initial staging of Non‐Small‐Cell Lung Cancer (NSCLC)^6^ and for providing functional information in treatment planning.^7^ The use of Standardized Uptake Values (SUV) has become more commonly accepted in clinical FDG‐PET oncology imaging, and it has found a specific role in assessing patient response to cancer therapy.^8^, ^9^

Stereotactic body radiotherapy (SBRT) is an effective treatment for early‐stage NSCLC and has been predominately used for medically inoperable patients. Most cancer centers in the United States follow guidelines based on Radiation Therapy Oncology Group (RTOG) protocols to evaluate lung SBRT treatment planning^10^ and to assess clinical outcomes in patient follow‐up.^11^ Computed Tomography (CT) is used as a mandatory follow‐up to assess tumor response after lung SBRT. As an example, RTOG requires CT scans every 3 months during the first 2 years after SBRT; then every 6 months for 2 more years to assess outcomes. 18F‐FDG PET scanning was used only if CT scans showed progressive soft tissue abnormalities.^12^ Some cancer centers also use 18F‐FDG PET/CT (simplified as PET/CT) for follow‐up. There have been some pilot trials and studies using 18F‐FDG PET to predict treatment outcomes before lung SBRT^13^, ^14^, ^15^ or assess clinical outcomes after lung SBRT.^16^, ^17^, ^18^, ^19^, ^20^, ^21^, ^22^, ^23^, ^24^ In this study we retrospectively investigated tumor responses of lung SBRT patients for different SBRT prescriptions, analyzed the relation between optimal biologically equivalent dose (BED) and tumor responses for SBRT prescriptions.

## 2 MATERIALS AND METHODS

### 2.A Lung SBRT patients and treatment

In our clinics pre‐SBRT evaluations consisted of history and physical examinations, contrast‐enhanced CT scans of the chest, PET/CT scans, and pulmonary function testing. Biopsies were performed unless medically contraindicated. Regular follow‐ups with CT imaging were performed on all patients. Post‐SBRT PET/CT was not mandated and was typically ordered at the discretion of the treating physician and upon concern for disease recurrence. For four SBRT prescriptions (9 Gy × 5, 10 Gy × 5, 11 Gy × 5, and 12 Gy × 4) historically recommended by RTOG there were 48 patients with documented PET/CT scans approximately 3 months after SBRT completion, among 102 SBRT patients treated between 2010 and 2016. 10 patients had two or more post‐SBRT PET/CT scans at a median of 8 months.

All SBRT patients were treated on 21iX and Trilogy machines (Varian Medical Systems, Palo Alto, CA, USA) using RapidArc^®^ (VMAT) Radiotherapy Technology guided with Cone Beam Computed Tomography (CBCT) and orthogonal images. Certain details of our lung SBRT have previously been reported.^25^ All SBRT plans met evaluation criteria and normal tissue constraints derived from recent RTOG protocols and the AAPM TG report^26^ including percentage of Planning Tumor Volume (PTV) covered by prescription dose: PTV_100%_ ≥ 90% and PTV_90%_ ≥ 99%; ratio of prescription isodose covered volume to PTV: R_100%_ < 1.2; and percentage of total lung receiving more than 20 Gy dose: *V*_20 Gy_ < 10%.

### 2.B Tumor response assessment

PET/CT scans were performed on a GE Discovery PET/CT Scanner (GE Healthcare, Chicago, IL, USA). The patients received 10–20 mCi of 18F‐FDG 45–60 min before imaging. PET images were reconstructed in 3D (axial, sagittal, and coronal) views and automatically registered with CT images. Physicians reviewed the fused PET/CT images and read the max SUV in a Region of Interest (ROI) around the tumor on the PET images. While reading post SUVs, ROIs should be carefully selected based on fused CT images, since PET images will not show the tumors successfully treated. All max SUVs were obtained from reviewing charts. Some max SUVs were re‐evaluated using the GE workstation. Patients who had PET scans without documented SUVs were excluded.

Tumor response assessment using 18F‐FDG uptake PET relies on four primary criteria,^27^ namely Complete Metabolic Response (CMR), Partial Metabolic Response (PMR), Stable Metabolic Disease (SMD), and Progressive Metabolic Disease (PMD). This study used simplified tumor response criteria: (a) Complete Response (CR) − post max SUV (SUV_post_) after SBRT in the treated tumor region was almost the same as the SUVs in the surrounding regions; (b) Partial Response (PR) − SUV_post_ was smaller than previous max SUV (SUV_pre_), but was greater than the SUVs in the surrounding regions; (c) No Response (NR) − SUV_post_ was the same as or greater than SUV_pre_. Some SUV_post_ reported as mild or favorable responses was classified as CR/PR.

### 2.C Biologically equivalent dose

Biologically equivalent dose (BED) has been introduced into optimal doses and fractionation schedules of SBRT.^28^, ^29^, ^30^, ^31^, ^32^ BED_10_, BED calculated using *α*/*β* of 10 Gy in the linear quadratic model, is used to predict of local control in lung SBRT.

(1) $$BED=D\times \left(1+\frac{d}{\frac{\alpha }{\beta }}\right)$$

where D: total dose; d: fractional dose; and α/β: ratio of the linear quadratic model when combined with clinical data on the steepness of the dose–response curve.

## 3 RESULTS

### 3.A SBRT planning and tumor response from PET/CT

**Figure 1:**
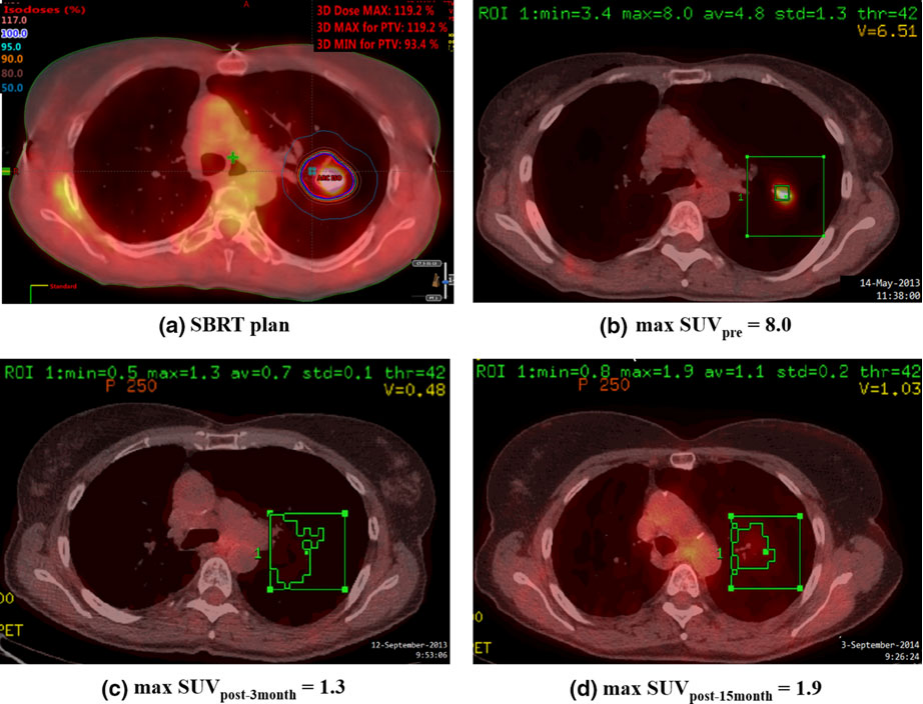
(a) 11 Gy × 5 SBRT plan; (b) PET/CT before SBRT, SUV_pre_ = 8.0; (c) PET/CT 3 months after SBRT, SUV_post‐3 month_ = 1.3; (d) PET/CT 15 months after SBRT, SUV_post‐15 month_ = 1.9. Green boxes were ROIs sampling SUVs (minimum, maximum, average, and standard deviation).

Figure 1(a) shows a typical SBRT RapidArc^®^ plan for a typical early‐stage NSCLC. 11 Gy × 5 was prescribed to PTV (20.7 cc). Target coverage: V_100%_ ≥ 90% and V_90%_ ≥ 100%; dose conformity: R_100%_ = 1.0; and *V*_20 Gy_ = 5.3%. The plan met all evaluation criteria and normal tissue constraints listed in RTOG 0813, and the patient completed treatment within 2 weeks. Fig. 1(b) shows the PET/CT scan before SBRT with SUV_pre_ = 8.0. Fig. 1(c) and 1(d) show PET/CT images taken 3 and 15 months after SBRT respectively. Both SUV_post‐3 month_ and SUV_post‐15 month_ were the same as SUVs in the surrounding regions.

After reviewing all charts and re‐evaluating some SUVs on the PET/CT images, we observed that max SUV_post_ at or below 1.9 showed similar max SUVs in surrounding regions for patients treated with SBRT in this study. Tumor response for these patients was classified as CR after SBRT. Some SUV_post_ ≤ 2.5 was indicated as mild or favorable responses in some PET/CT diagnosis reports, we classified 1.9 < SUV_post_ ≤ 2.5 as CR/PR.

### 3.B Tumor response over time

**Figure 2:**
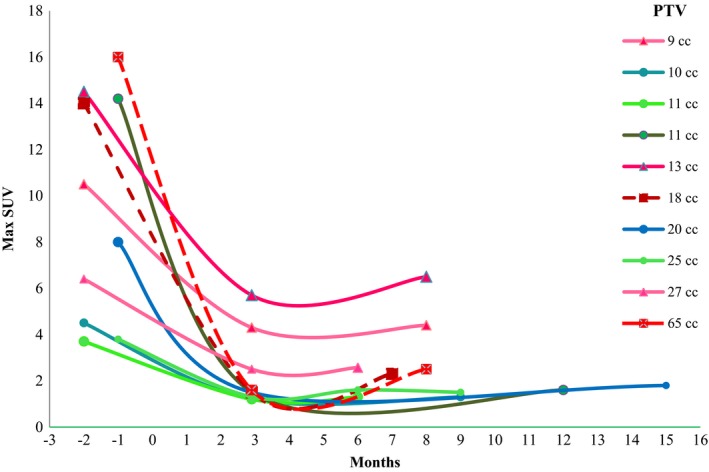
Max SUV trends over time for 10 patients with more than two PET/CT scans after SBRT completion. SBRT completion was defined as month zero for each treatment. Max SUV values were connected with smoothed lines for each patient. PTVs were listed for the patients.

Ten patients (median PTV, 15.1 cc; range, 9.4−65.3 cc) had two or more PET/CT scans after SBRT completion. Before SBRT the median SUV_pre_ was 12.3 (range, 3.7−16.0). After SBRT the median of the 1^st^ SUV_post_ was 1.5 (range, 1.2−5.7) at a median time of 2.9 months (range, 2.3−3.5 months). The median of the 2^nd^ SUV_post_ was 2.1 (range, 1.3−6.5) at a median time of 8 months (range, 6−15 months). The max SUV values over time for these 10 patients are plotted in Fig. 2.

In Fig. 2 the *x*‐axis indicates the month before or after SBRT completion in which max SUV values were read. SUV values were plotted with different patterns for three groups: (a) rounded − both 1^st^ SUV_post_ and 2^nd^ SUV_post_ were at or below 1.9; (b) triangle − 1^st^ SUV_post_ and 2^nd^ SUV_post_ were both above 1.9 and were similar values; (c) square − 2^nd^ SUV_post_ was greater than 1^st^ SUV_post_ and above 1.9. There were five patients in group (a): median PTV was 11 cc (range, 10−25 cc) and median max SUV_pre_ was 4.5 (range, 3.7−14.2). For these five CR cases we observed PTVs were relatively small; however, max SUV_pre_ was as high as 14.2. There were two patients in group (c): PTVs were 18 cc and 65 cc and respective max SUV_pre_ values were 14.0 and 16.0. For these two cases we observed that PTVs were relatively large with high max SUV_pre_ values. We found that all 1^st^ SUV_post_ values were smaller than 2^nd^ SUV_post_ values. The 1^st^ SUV_post_ was read at a median of 2.9 months after SBRT completion (range, 2.3−3.5 months) in this study.

### 3.C Tumor response for different prescriptions

For patients treated with these four prescriptions (9 Gy × 5, 10 Gy × 5, 11 Gy × 5, and 12 Gy × 4), the median PTVs were 14.8 cc, 11.1 cc, 17.3 cc, and 8.1 cc; the median SUV_pre_ values were 7.6, 5.8, 4.5, and 4.7; and the median SUV_post‐3 month_ values were 2.0, 2.2, 1.9, and 1.4, respectively, as listed in Table 1.

**Table 1:** Max SUV for different SBRT prescriptions

# Pts	SBRT	PTV (cc)		SUV_pre_		SUV_post‐3 month_	
	Prescription	Median	Range	Median	Range	Median	Range
7	9 Gy×5	14.8	6.6–65.3	7.6	2.5–20.0	2.0	1.0–5.4
15	10 Gy×5	11.1	2.8–57.7	5.8	3.5–14.7	2.2	1.4–11.0
12	11 Gy×5	17.3	6.1–25.3	4.5	3.0–14.2	1.9	1.4–6.6
14	12 Gy×4	8.1	2.5–16.6	4.7	1.5–14.5	1.4	1.2–11.9

# Pts, Number of Patients; SBRT, Stereotactic body radiotherapy; PTV, Planning Tumor Volume; SUV_pre_, max Standardized Uptake Value pre SBRT; SUV_post‐3 month_, max Standardized Uptake Value 3 months after SBRT.

Among these 48 SBRT patients with documented SUV_post‐3 month_, there were 3 NR cases (6%); 8 PR cases (17%); 37 CR and CR/PR cases (77%). Of these CR and CR/PR cases, there were 26 CR cases (54%).

**Figure 3:**
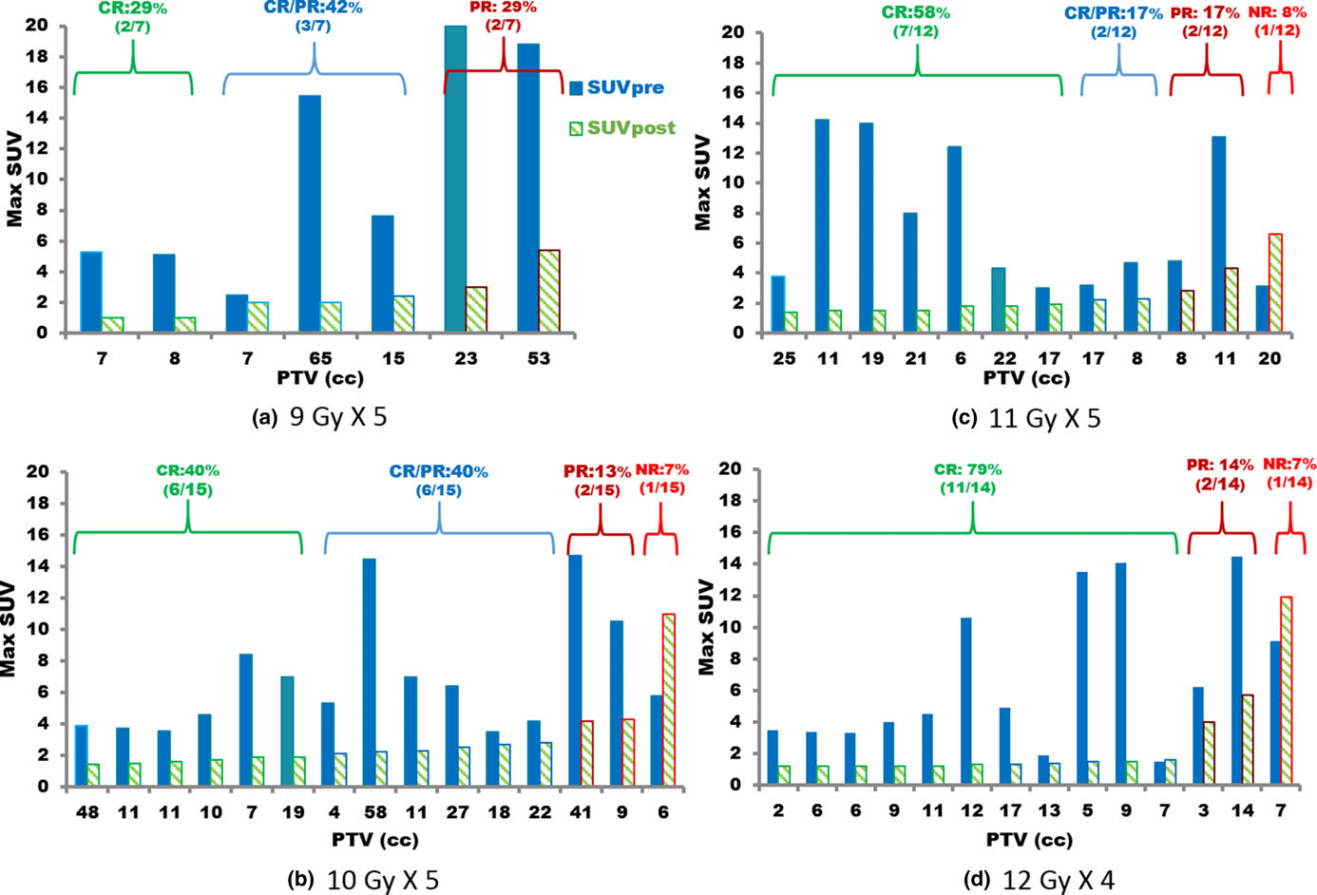
Max SUV for different prescriptions: max SUVs (*y*‐axis) before SBRT (solid bars) and 3 months after SBRT (stripped bars). The *x*‐axis shows PTV (cc) and is sorted by SUV_post‐3 month_ for each prescription: (a) 9 Gy × 5; (b) 10 Gy × 5; (c) 11 Gy × 5; and (a) 12 Gy × 4.

Max SUVs are plotted in Fig. 3. Tumor responses were classified into four categories: (a) CR − SUV_post‐3 month_ was at or below 1.9; (b) CR/PR − SUV_post‐3 month_ was between 2.0 and 2.5, inclusive; (c) PR − SUV_post‐3 month_ was at or above 2.6 but smaller than SUV_pre_; and (d) No NR − SUV_post‐3 month_ was the same as or greater than SUV_pre_. For the 9 Gy × 5 prescription, the percentages of CR, CR/PR, PR, and NR were 29%, 42%, 29%, and 0%, respectively; for 10 Gy × 5, the percentages were 40%, 40%, 13%, and 7%; for 11 Gy × 5, the percentages were 58%, 17%, 17%, and 8%; and for 12 Gy × 4, the percentages were 79%, 0%, 14%, and 7%.

Table 2 shown BED_10_ were 85.5 Gy, 100.0 Gy, 115.5 Gy, and 105.6 Gy; and relevant complete response rate (CR) based on max SUV_post‐3 month_ were 29%, 40%, 58%, and 79% corresponding to 9 Gy × 5, 10 Gy × 5, 11 Gy × 5, and 12 Gy × 4 respectively.

**Table 2:** Tumor response for different SBRT prescriptions

Prescription	PTVave (cc)	BED_10_ (Gy)	CR (%)	CR/PR (%)	PR (%)	NR (%)
9 Gy × 5	25.5	85.5	29	42	29	0
10 Gy × 5	21.1	100.0	40	40	13	7
11 Gy × 5	15.5	115.5	58	17	17	8
12 Gy × 4	6.8	105.6	79	0	14	7

## 4 DISCUSSIONS

### 4.A Why/how do we use max SUV from PET to assess tumor responses?

Assessment of tumor response in cancer therapeutics is a multidisciplinary task.^33^ CT is used as a mandatory follow‐up to assess tumor local control, recurrence, and distant metastasis after radiotherapy. Using CT, it may require more than 2 years to completely assess tumor responses,^34^ and so CT alone may be unable to accurately assess tumor response early enough to allow for some salvage treatment modalities.^9^ PET measures biochemical changes using 18F‐FDG rather than evaluating tumor size differences from images as CT does,^9^, ^35^ and so PET is able to assess tumor response sooner than CT. PET/CT combined a max SUV (a quantitative measure of 18F‐FDG accumulation) read from PET and an ROI (a region covering the tumor) defined from CT, has become an important tool in assessing tumor response.

Using SUV_post‐3 month_ to assess the tumor responses was based on several considerations. First, most post PET/CT scans were taken around 3 months after SBRT. Second, all 2^nd^ SUV_post_ were either similar or slightly greater to the readings of SUV_post‐3 month_ in this study. Third, 50% (5 out of 10) patients had both SUV_post‐3 month_ and 2^nd^ SUV_post_ at or below 1.9 as shown in Fig. 2. Henderson et al.^19^ collected post max SUVs from PET/CT scans at 2, 26, and 52 weeks after SBRT. The trend of max SUV over time in their 14 patients study had similar results as shown in Fig. 2. Max SUV taken around 3 months after SBRT completion has also been applied to assess tumor responses in recent studies.^23^, ^24^

In this study we used a SUV_post_ at or below 1.9 as CR criteria. It should be noted that this value only applies to our clinic. SUV depends on patient size and the amount of injected FDG, as well as the duration of the uptake time between FDG injection and scan start.^8^ Even if the same PET/CT scanning protocol was followed, there could still be some variability introduced by differing calibrations of scanners and performing readings on different workstations. However, each clinic should have some thresholds of SUV_post_ for different tumor responses.

CT/X‐ray imaging were performed as the regular follow‐up on all patients at our clinic. Post‐SBRT PET/CT usually ordered at the discretion of the treating physician. Biopsies were also performed for confirming tumor recurrences. Table 3 compared the PET tumor responses defined in this study to the patient follow‐ups based on the CT/X‐ray imaging and biopsy data. Local Control (LC) is defined as a tumor shrinkage shown in the CT/X‐ray images after SBRT, Regional Failure (RF) is defined as an enlarging nodule adjacent to the treated area observed on the CT images, and all tumor recurrences were confirmed by biopsies before re‐treatments. Although our SBRT program followed RTOG protocols’ prescriptions and normal tissue constraints, the patient follow‐up did not follow any protocol. The individual follow‐up interval and period varied on each patient. All the patients in this study had 1‐year CT/X‐ray imaging follow‐ups, while only 23 (48%) patients had documented 2‐year or longer follow‐ups. In Table 2, 3‐year^a^ was the follow‐up time documented in the period of 2–5 years after SBRT.

**Table 3:** PET Tumor response vs patient follow‐up

Tumor response	# Pts	1‐year LC	1‐year RF	2‐year^a^ RF	2‐year^a^ OS	# Re‐TX
CR	26	26 (100%)	0	1	25	1
CR/PR	11	11 (100%)	0	3	11	3
PR	8	8 (100%)	4	1	8	5
NR	3	0 (0.0%)	3		3	3
Total	48		7	5	47	12

# Pts, Number of Patients; LC, Local control; RF, Regional Failure; OS, Overall Survival; # Re‐TX, Number of patients with recurrent tumor treated at the same site of previous SBRT.

a documented in the period of 2–5 years after SBRT.

Table 3 shown there was only one (4%) RF after 2 years follow‐up for 26 CR cases; 3 (27%) RF after 2 years for 11 CR/PR cases; 5 (63%) RF after 2 years for 8 PR cases; and 3 (100%) RF within one year for 3 NR cases. Confirmed by biopsy all the RF tumors were re‐treated. More than 70% (8 over 11) CR/PR cases had the similar follow‐up results as the CR cases. It worth to note the RF tumor in CR category initially had a good response, but then developed an enlarging mass; initially was biopsy negative for malignancy, but ultimately was biopsy confirmed recurrent disease. It also should be noted there was only one 80‐year old patient expired in these selected 48 patients. This patient initially had a favorable tumor response after 9 Gy×5 SBRT, ultimately expired due to his Stage IV lung cancer in 3 years after SBRT.

The regular patient follow‐ups confirmed 3‐month post max SUV could accurately assess tumor responses after lung SBRT.

### 4.B What is an optimal prescription for a lung SBRT patient?

In Table 2 we observed that higher BED_10_ and lower tumor volume would achieve higher complete response (tumor control) rates. The highest complete response rate was observed for smallest tumor volume (PTV_ave_ = 6.8 cc) with higher BED_10_ (105.6) of 12 Gy × 4 prescription. For 11 Gy × 5 prescription, the BED_10_ (115.5) was the highest in these four groups, but its complete response rate (58%) was lower than 79% of 12 Gy × 4 prescription. We found the average tumor volume of 11 Gy × 5 group was more than double of the volume of 12 Gy × 4 group. It worth to note the current linear quadratic model to predict tumor control using BED [Eq. (1)] does not consider tumor volume. A recent study^32^ also mentioned the escalation of BED to high levels (>150 Gy) would be required for patients with a tumor size >3 cm in lung SBRT. Modification of the linear quadratic model and dose escalation for patients with larger tumor volume were necessary.

Table 4 listed the tumor characteristics treated with 12 Gy × 4 SBRT (BED_10_ = 105.6). All the tumors were peripheral lung lesions. 10 were Stage IA tumors, three were Stage III, and one was Stage IV. All Stage IA tumors had CR after the SBRT; One Stage IIIA tumor, which had two nodules at different lobes in the same lung, also had CR, the other two Stage III tumors only got PR; The Stage IV tumor, which was initial from Stage IIA of colon cancer, did not response to the SBRT.

**Table 4:** Tumor characteristics vs tumor responses after 12 Gy × 4 SBRT (BED_10_ = 105.6)

Case #	SITE	Vol (cc)	STAGE	SUV_pre_	SUV_post‐3 month_	Response
1	RUL	2.47	IA T1a N0	3.5	1.2	CR
2	RUL	5.23	IA T1a N0	13.5	1.5	CR
3	LUL	5.72	IA T1a N0 M0	3.4	1.2	CR
4	LLL	6.19	IA T1a N0	3.3	1.2	CR
5	RLL	7.35	IA T1 N0 M0	1.5	1.6	CR
6	RUL	8.82	IA T1a N0	14.1	1.5	CR
7	LUL	8.51	IA T1a N0	4.0	1.2	CR
8	RUL	10.66	IA T1 N0 M0	4.5	1.2	CR
9	RUL	13.08	IA T1a N0	1.9	1.4	CR
10	RUL	16.60	IA T1 N0 M0	4.9	1.3	CR
11	RUL	12.27	IIIA T4 N0 M0^a^	10.6	1.3	CR
12	RUL	3.22	IIIB T4 N3 M0	6.2	4.0	PR
13	RUL	13.64	IIIA T2a N0 M0	14.5	5.7	PR
14	RUL	6.91	IV^b^	9.1	11.9	NR

Case #, Tumor labeling; SUV_pre_, previous max SUV before SBRT; SUV_post‐3 month_, max SUV 3 months after SBRT; RUL, Right Upper Lobe; LUL, Left Upper Lobe; LLL, Left Lower Lobe; RLL, Right Lower Lobe.

a 2 nodules, at different lobes, in the same lung.

b initial from Stage IIA of colon cancer.

The data in Table 4 shown the SBRT prescription (BED_10_ > 100) could get very favorable tumor response for early staging lung cancer, but BED_10_ should be escalated for late staging tumor, thus the linear quadratic model to predict tumor control should also include tumor staging.

## 5 CONCLUSION

We defined a tumor response criterion using post max SUV from PET/CT taken around 3 months after treatment to assess the tumor responses for lung SBRT using RapidArc^®^ technique. We demonstrated post max SUV read from PET/CT could efficiently and accurately assess tumor response after lung SBRT. We suggest 3‐month post max SUV read from PET/CT should become standard tumor response assessment for lung SBRT. Although SBRT with prescriptions resulting in a BED_10_ > 100 experienced favorable tumor responses for early staging non‐small cell lung cancer (NSCLC), escalation of BED_10_ to higher levels may be beneficial for lung cancer patients with later staging and larger volume tumors.

## CONFLICTS OF INTEREST

The authors do not have any conflicts of interest to declare.
